# Long noncoding HOXD-AS1: a crucial regulator of malignancy

**DOI:** 10.3389/fcell.2025.1543915

**Published:** 2025-03-26

**Authors:** Xiang-Yuan Tao, Qian-Qian Li, Shan-Shan Dong, Hui Wang, Yu-Qing Yang, Xi Yang, Yong Zeng

**Affiliations:** ^1^ School of Pharmaceutical Science, Hengyang Medical School, University of South China, Hengyang, China; ^2^ Translational Medicine Center, Hunan Cancer Hospital/The Affiliated Cancer Hospital of Xiangya School of Medicine, Central South University, Changsha, China

**Keywords:** lncRNA, HOXD-AS1, cancer, molecular mechanisms, potential clinical value

## Abstract

Long non-coding RNAs (lncRNAs) play a crucial role in the occurrence and progression of various cancers. HOXD-AS1, an antisense RNA 1 of the lncRNA HOXD cluster, (also known as HAGLR, MIR7704HG, Mdgt, and STEEL), is located at human chromosome 2q31.1. Recent studies have demonstrated that the abnormal expression of HOXD-AS1 is significantly correlated with the clinicopathological features of patients with various tumors. The expression of HOXD-AS1 is abnormal in various tumors, affecting tumor cell proliferation, apoptosis, metastasis, invasion, metabolism, and drug resistance. HOXD-AS1 is important for cancer diagnosis and prognosis evaluation. Detecting its expression level helps judge cancer progression and predict patient survival. It is a therapeutic target and biomarker for early diagnosis and prognosis, with good clinical application prospects. This article reviews the role, molecular mechanisms, and potential clinical value of HOXD-AS1 in malignant tumor development.

## 1 Introduction

Cancer treatment remains a global healthcare challenge. Future global cancer incidence rates are expected to keep rising from 2020 to 2040 (Xie et al., 2023; Siegel et al., 2023a; Jassim et al., 2023). Cancer is a genetic disease caused by various driving forces and influencing factors such as epigenetics, genetic disorders, and environmental elements. Currently, there are limitations in cancer treatment. Thus, further understanding the pathogenesis of cancer and identifying related targets and prognostic markers is crucial for cancer treatment (Fusco et al., 2021; Ahles and Root, 2018). In addition to protein-coding genes, mutations and abnormal expression of non-coding RNA, especially lncRNAs, also play a vital role in cancer (Chakravarty and Solit, 2021; Sanfilippo and Hewitt, 2014). lncRNAs act as transcriptional, chromatin, and post-transcriptional regulators (Gong et al., 2022; Ponting et al., 2009). Antisense lncRNA is produced in the opposite direction of transcription of protein-coding transcripts and is widely found in eukaryotes (Faghihi and Wahlestedt, 2009). The lncRNA HOXD cluster antisense RNA1(HOXD-AS1) in various cancers is related to tumor occurrence and development, providing a new direction for the clinical management of tumor patients. This article reviews the complex molecular pathways and mechanisms of HOXD-AS1 in various malignant tumors and the correlation between HOXD-AS1 expression and tumor size, depth of invasion, tumor differentiation, tumor stage, lymph node metastasis, and recurrence (Table 1). It aims to summarize the role of HOXD-AS1 in tumorigenesis and its potential in cancer prediction, diagnosis, and prognosis.

**TABLE 1:** The biological function and regulatory mechanism of HOXD-AS1 in digestive system tumors.

Cancer types	Expression	Sample type	Clinical features	Biological functions	Related genes and pathways	References
Hepatocellular carcinoma	Upregulated	Cell lines	Overall survival, Tumor volume, TNM stage	Proliferation (+), Migration (+), Invasion (+), Migration (+)	miR-19a/ARHGAP11A, miR-130a-3p/SOX4, miR-326/SLC27A4	Wang et al. (2017a), Lu et al. (2017), Ji et al. (2020), Sun et al. (2020)
Colorectal cancer	Upregulated Downregulation	Cell lines	Overall survival and disease-free survival, metastasis, Differentiation Distant metastasis TNM stage Pathological grades	Proliferation (+), migration (+), Invasion (+), Stemness (+) Proliferation (+), migration (+), Invasion (+)	miR-217/AIG-1, miR-526b-3p/CCND1	Li et al. (2018b), Yan et al. (2020)
Gastric cancer	Upregulated	Tissues and cell lines	invasion depth TNM stages lymphatic metastasis regional lymph nodes distant metastasis Overall survival	Proliferation (+), Drug resistance (+)	miR-338-3p/JAK2/STAT3	Hu et al. (2022), Zheng et al. (2017)
Oral squamous cell carcinoma	Upregulated	Cell lines	Overall survival	Migration (+), Invasion (+)	miR-203a-5p/Annexin A4	Zhang et al. (2023)
Cholangiocarcinoma	Upregulated	Tissues and cell lines	Overall survival Lymph node tumor volume, invasion metastasis, TNM stage	Malignant progression (+)	SP1/miR-520c-3p/MYCN	Li et al. (2020)
Pancreatic Cancer	Upregulated	Cell lines	Overall survival Tumor-Node-Metastasis stage	Proliferation (+), Migration (+), Invasion (+)	miR-664b-3p/PLAC8	Chen et al. (2022)

## 2 Overview of HOXD-AS1

lncRNA HOXD cluster antisense RNA1(HOXD-AS1) is transcribed from the HOXD gene cluster situated on human chromosome 1q31.1 and is distributed in both the nucleus and cytoplasm (Yang et al., 2019). The human HOX gene can be divided into A, B, C, and D gene clusters, each containing 9 to 11 genes. These are conserved genes located on different chromosomes, and abnormal expression is associated with malignancy. HOXD belongs to the homeobox gene and is located on human chromosome 2q31.1. It plays a significant role in early embryonic development and tissue morphogenesis. Its mutation can lead to fetal malformation and functional abnormalities (Su et al., 2018; Johnston et al., 1998; Gehring and Hiromi, 1986). In a meta-analysis study based on the TCGA database, among approximately 9,502 cancer patients from more than 30 cancer types, the group with high HOXD-AS1 expression was associated with shorter overall survival (OS, *P* = 0.0019) and disease-free survival (DFS, *P* = 0.00013) (Zhang et al., 2018). In addition, HOXD-AS1, as a new lncRNA, is associated with clinicopathological features such as stage, TNM stage and lymph node metastasis of various tumors (Li L. et al., 2018). The NCBI database reveals that HOXD-AS1 is expressed in various tissues, with the kidney, colon, and testis showing the highest expression level. HOXD-AS1 correlates with the gene expression of HOXD1 and HOXD3. In recent years, an increasing number of studies have indicated that HOXD-AS1 is dysregulated in cancer. Its high expression is related to tumor cell proliferation, migration, and invasion, as well as the inhibition of apoptosis, which accelerates the occurrence and development of tumors (Xie et al., 2019). Therefore, understanding the role of HOXD-AS1 in malignancy and its mechanism of action is critical.

## 3 The role of HOXD-AS1 in the digestive system tumors

### 3.1 Hepatocellular carcinoma

Hepatocellular carcinoma (HCC) is the most common type of primary liver cancer, with insidious onset, slow disease development and high recurrence rate. Kaplan-Meier analysis demonstrated that high levels of HOXD-AS1 were associated with a lower overall survival rate in HCC patients (*P* = 0.0179). Multivariate regression analysis revealed that high expression of HOXD-AS1 was an independent and significant factor affecting OS (HR: 0.552; 95% CI: 0.321–0.950; *P* = 0.032). Wang et al. found that the upregulation of HOXD-AS1 was significantly correlated with the advanced Tumor Node Metastasis (TNM) stage of HCC patients in 120 HCC patients with follow-up data (Wang H. et al., 2017). In addition, in hepatocellular carcinoma, HOXD-AS1 is enriched in the cytoplasm. Its increased expression promotes the proliferation and migration of hepatocellular carcinoma cells. Since the expression of HOXD-AS1 can also be localized in the nucleus or cytoplasmic components, it can be hypothesized that HOXD-AS1 may play a role in chromatin remodeling Since the expression of HOXD-AS1 can also be localized in the nucleus or cytoplasmic components, it can be speculated that HOXD-AS1 may play a role in chromatin remodeling. In terms of mechanism of action, Wang et al. found that transcription factor STAT3 can bind to the promoter of HOXD-AS1 and activate the transcription of HOXD-AS1, and HOXD-AS1 can bind to miR-130a-3p through ceRNA mode in HCC, preventing SRY-related HMG-box 4 (SOX4) from being degraded by miRNA, thereby activating the expression of Enhancer of Zeste Homolog 2 (EZNH2) and Matrix Metalloproteinase 2 (MMP2) and promoting HCC metastasis (Wang H. et al., 2017). Concurrently, it was also observed that HOXD-AS1 significantly reduces the apoptotic effect by downregulating the expression of RGS3, a potential inhibitor of the MEK-ERK1/2 signaling axis (Wang H. et al., 2017; Lu et al., 2017; Ji et al., 2020; Sun et al., 2020). In addition, HBV (Hepatitis B Virus) and HCV (Hepatitis C Virus) infections are important causes of HCC. Although no studies directly link HOXD-AS1 expression to HBV or HCV, some research shows associations between certain lncRNA transcripts and HBV. For example, upregulated lncRNA MAPKAPK5-AS1 can enter HBV-positive HCC cells *via* exosomes and promote their proliferation by targeting the MYC proto-oncogene (c-Myc) (Tao et al., 2022). Similarly, HBV protein X (HBx) in HBV-positive HCC inhibits lncRNA OIP5-AS1 expression, acting as a tumor promoter and a potential diagnostic and therapeutic target (Shi et al., 2024). Also, lncRNA PCNAP1 can trigger liver cancer by regulating the miR-154/PCNA/HBV pathway (Feng et al., 2019). HOXD-AS1 is similar to these lncRNAs, and its abnormal expression affects HCC cell proliferation and migration. So, we think HBV may influence HOXD-AS1 expression, possibly leading to HCC development. To sum up, these studies have indicated that overexpression of HOXD-AS1 can enhance the proliferation, migration, invasion, and epithelial-mesenchymal transition of hepatocellular carcinoma cells and inhibit apoptosis. This implies that HOXD-AS1 may be a promising therapeutic target for the treatment of HCC. However, its specific carcinogenic mechanism still needs further exploration.

### 3.2 Colorectal cancer

Colorectal cancer (CRC) ranks as the second most common cause of cancer-related deaths in the United States, Moreover, CRC is increasingly being diagnosed at a younger age and in a more advanced stage (Siegel et al., 2023b; Guan et al., 2023; Li X. et al., 2018). The high expression of HOXD-AS1, when it is localized in the nucleus, is regulated by the HOXD-AS1-HOXD3-Integrinβ3 pathway. A low expression of HOXD-AS1 in the nucleus can promote tumor cell proliferation and metastasis. Additionally, a low level of HOXD-AS1 expression is significantly associated with a poor prognosis, low differentiation (*P* = 0.047), advanced TNM stage (*P* < 0.05), and low survival rate (*P* < 0.05) among colorectal cancer patients (Yang et al., 2019). In terms of mechanism, HOXD-AS1 can function as a competitive endogenous RNA of miR-217. Knockdown of HOXD-AS1 can inhibit cell proliferation, invasion, epithelial-mesenchymal transition, and stem cell formation *in vitro*, and also inhibit tumor growth and metastasis *in vivo* (Li X. et al., 2018). Furthermore, studies have discovered that HOXD-AS1 can target miR-526b-3p. Consequently, the inhibitor of miR-526b-3p and its target gene CCDN1 can reverse the inhibitory effect of HOXD-AS1 on the proliferation, migration, and invasion of colorectal cancer cells (Yan et al., 2020). The above studies have shown that different localizations of HOXD-AS1 can regulate the biological behavior of tumor cells through different signaling pathways and influence the occurrence and development of diseases. In conclusion, most of the existing research focuses on the mechanism of HOXD-AS1 in the cytoplasm. In other diseases, whether HOXD-AS1 located in the nucleus has corresponding biological functions, its specific mechanism of action, and its relationship with disease diagnosis and treatment are worthy of further investigation.

### 3.3 Gastric cancer

Gastric cancer (GC) ranks as the fifth most common malignant tumor globally and is the fourth leading cause of cancer-related deaths (Guan et al., 2023; Sung et al., 2021). According to clinical data, high expression of HOXD-AS1 is remarkably associated with larger tumors, deeper infiltration, advanced TNM stage, regional lymph node metastasis, lower patient survival rate, and distant metastasis (*P* < 0.05) (Hu et al., 2022). Zheng et al. found that HOXD-AS1 was upregulated in GC tissues as well as in GC cell lines such as SGC-7901 and BGC-823. The high expression of HOXD-AS can drive cell growth by activating signaling pathways related to JAK2 and STAT3 target genes (Zheng et al., 2017). Jing Hu et al. found that HOXD-AS1 can promote the resistance of GC cell lines to 5-FU by binding to miR-338-3p and targeting the lactate dehydrogenase A (LDHA) -glycolysis pathway (Hu et al., 2022). To sum up, although existing studies have uncovered that HOXD-AS1 can facilitate drug resistance in gastric cancer by modulating related genes through the ceRNA mode, there remains a lack of further in-depth examination of its upstream signaling pathways and downstream specific molecular regulatory networks.

### 3.4 Oral squamous cell carcinoma

Oral squamous cell carcinoma (OSCC) constitutes approximately 90% of oral malignancies. According to the Global Cancer Observatory (GCO), the incidence of OSCC is expected to increase by around 40% by 2040, accompanied by a corresponding increase in mortality (Tan et al., 2023). Padam et al. constructed an interaction network to analyze the non-coding RNA in the HOX cluster and discovered that HOXD-AS1 affected the prognosis and overall disease survival of patients with oral cancer patients, further confirming that the high expression of HOXD-AS1 is associated with the occurrence and development of oral cancer (Padam et al., 2022). Zhang et al. used reverse transcription-polymerase chain reaction (RT-qPCR) to verify the expression of HOXD-AS1 in cancer and non-tumor tissues of 60 patients with oral squamous cell carcinoma. They found that patients in the high-level group (*n* = 30) had a poorer prognosis than those in the low-level HOXD-AS1 group (*n* = 30). In the oral squamous cell carcinoma cell line SCC25, HOXD-AS1 regulates the Annexin A4-related signaling pathway by competitively binding to miR-203a-5p, promoting cell migration and invasion. This study demonstrated for the first time that HOXD-AS1 may act as an endogenous transcription factor (Zhang et al., 2023). It is worth noting that although studies have shown that HOXD-AS1 has a cancer-promoting role in OSCC, there is a lack of verification at the clinical and animal levels. Moreover, the relationship between HOXD-AS1 and the clinicopathological characteristics and survival prognosis of OSCC patients still needs to be further explored.

### 3.5 Cholangiocarcinoma

Cholangiocarcinoma (CCA) is a malignant tumor of the digestive system originating from bile duct epithelium, which has a very poor prognosis (Calvisi et al., 2023). In recent years, research has revealed that HOXD-AS1 is upregulated in the tissues and cells of patients suffering from CAA. Furthermore, HOXD-AA1 is significantly associated with lymph node metastasis, advanced TNM stage, and poor prognosis in CAA patients. In addition, it is discovered in the mechanism that HOXD-AS1 is induced by the transcription factor SP1. w It competitively binds to miR-520c-3p to regulate the oncogene V-Myc Avian Myelocytomatosis Viral Oncogene Neuroblastoma Derived Homolog (MYCN) related signaling pathway, thereby promoting the proliferation, migration, invasion, and epithelial-mesenchymal transition (EMT) of tumor cells, maintaining the characteristics of stem cells, and reducing the sensitivity of cells to drugs. The SP1/HOXD-AS1/miR-520c-3p/MYCN axis plays a crucial role in the initiation and progression of CCA. Knockdown of HOXD-AS1 inhibited the volume and weight of tumor in nude mice, while co-transfection of miR-520c-3p partially reversed the inhibitory effect of HOXD-AS1 knockdown in nude mice. HOXD-AS1 is expected to be an effective biomarker and therapeutic target (Li et al., 2020).

### 3.6 Pancreatic cancer

**FIGURE 1:**
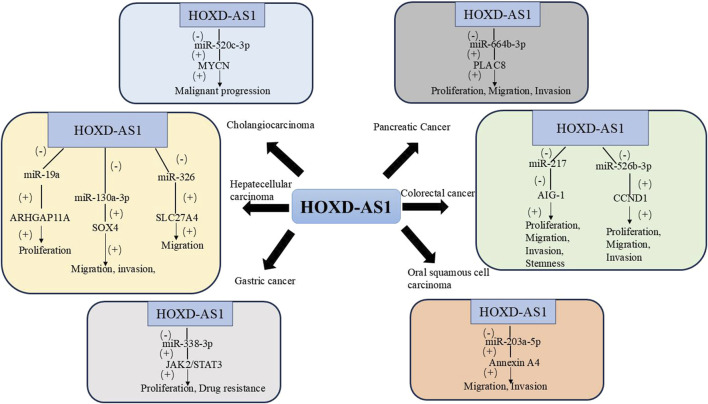
The regulatory network of HOXD-AS1 on digestive system tumors. (AIG-1, Apoptosis-Inducing Gene 1; ARHGAP11A, Rho GTPase activating protein 11A; CCND1, Cyclin D1; JAK2, Janus kinase 2; MYCN, V-Myc Avian Myelocytomatosis Viral Oncogene Neuroblastoma Derived Homolog; PLAC8, Placenta Specific Protein 8; SLC27A4, Solute Carrier Family 27 Member 4; SOX4, SRY-related HMG-box 4; STAT3, Signal Transducer and Activator of Transcription 3).

In recent years, the incidence of pancreatic cancer (PC) has been on the rise, and its 5-year survival rate is extremely low. Currently, there is no effective means for early diagnosis and treatment for PC, which is a malignant tumor with a poor prognosis (Halbrook et al., 2023; Zhao et al., 2023). Chen et al. found that the expression of HOXD-AS1 in PC tissues was significantly higher than that in adjacent tissues, and HOXD-AS1 was associated with advanced TNM staging. Mechanistically, HOXD-AS1 can upregulate Placenta Specific Protein 8 (PLAC8) by targeting mir-664b-3p and promote the proliferation, migration, and invasion of PC cells. This indicates that HOXD-AS1 may be a potential prognostic biomarker or therapeutic target for PC (Chen et al., 2022). Table 1 and Figure 1 summarizes the biological functions and regulatory mechanisms of HOXD-AS1 in digestive system tumors.

## 4 The role of HOXD-AS1 in genitourinary system tumors

### 4.1 Ovarian cancer

Most patients with ovarian cancer (OC) are diagnosed at a late stage. The case-fatality ratio of OC is three times that of breast cancer, making it the gynecological malignancy with the highest fatality rate. Therefore, to break free from this dilemma, it is of great significance to seek new treatment strategies (Kuroki and Guntupalli, 2020; Bray et al., 2018). In recent years, Dong et al. found that the expression of HOXD-AS1 was upregulated in OC tissues and OC cell lines SKOV3 and A2780, enhancing the migration, invasion, and EMT ability of cells. Among them, the validation of 200 clinical specimens showed that HOXD-AS1 regulated the PIK3R3 signaling pathway by competitively binding to miR-186-5p. The progression-free survival (PFS) and OS of patients with high expression of HOXD-AS1 were significantly reduced (Dong et al., 2019). In line with the results of this study, Wang and others discovered that HOXD-AS1 positively regulated the expression of frizzled family receptor 4 (FZD4) by competitively binding to miR-608, promoted the proliferation of OC cells, and enhanced the migration and invasion of OC cells. By analyzing the data of 369 OC patients in the TCGA database, it was shown that the PFS and OS of the HOXD-AS1 high expression group were lower. The above two studies have demonstrated that HOXD-AS1 may be a promising therapeutic target and a new prognostic marker for OC (Wang et al., 2018). Therefore, these research findings suggest that HOXD-AS1 in ovarian cancer may be involved in the proliferation and migration of tumor cells by interacting with multiple miRNAs and their respective target genes. In the ceRNA theory, HOXD-AS1 can serve as a cellular sponge to regulate the availability of specific miRNAs on the 3′untranslated region (UTR) of miRNA target genes. The above two studies have demonstrated that HOXD-AS1 may be a promising therapeutic target and a new prognostic marker for OC.

### 4.2 Cervical cancer

Cervical cancer (CC) ranks among the four major gynecological malignancies globally (Bray et al., 2018). The abnormal expression of HOXD-AS1 is associated with tumor lymph node metastasis stage, vascular invasion, lymph node metastasis, and recurrence. Knocking down HOXD-AS1 greatly inhibited cervical cancer cell proliferation *in vitro*. It also stopped tumor cell growth by inactivating the Ras/ERK signaling pathway. *In vivo* xenograft experiments confirmed these study results (Hu et al., 2017). HOXD-AS1 can upregulate the expression of fibroblast growth factor 2 (FGF2) through HOXD-AS1/miR-877-3p and regulate various malignant biological behaviors of CC cells (Chen and Li, 2020). The elevated expression of ferric chelate reductase 1 (FRRS1) is capable of promoting the proliferation and metastasis of tumor cells. Research has discovered that HOXD-AS1 can enhance the expression of FRRS1 by binding to ELF1 and influence the occurrence and development of cervical cancer (Liu et al., 2022). In addition, there’s no direct study on how HPV (Human Papilloma Virus) 16 and HPV18 regulate HOXD-AS1 in different cervical cancer cell lines. But studies found lncRNA MALAT1 is highly expressed in high-risk HPV-infected cervical cancer cell lines and tissues, promoting tumor cell proliferation, migration and invasion (Iordanishvili et al., 2023). The expression of lncRNA MEG3 was low in HPV16, HPV18 infection-related cervical cancer tissues and cervical cancer cell lines, suggesting that HPV infection may affect its expression (Zhang et al., 2016). An imbalance in HOXD-AS1 expression impacts cervical cancer cell proliferation and migration, like MALAT1 and MEG3 do in cervical cancer. So, we can infer that HPV may affect HOXD-AS1’s regulation and function, leading to cervical cancer development. The existing research findings have demonstrated the carcinogenic role of HOXD-AS1 in cervical cancer and put forward possible molecular mechanisms and signaling pathways. Nevertheless, more comprehensive studies are required to elaborate on downstream signal transduction and *in vivo* research to clarify its specific function in cervical cancer.

### 4.3 Prostate cancer

**FIGURE 2:**
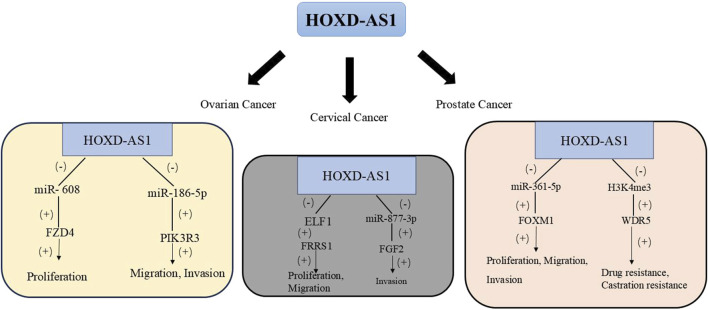
The regulatory network of HOXD-AS1 on genitourinary system tumors. (FGF2, fibroblast growth factor 2; FRRS1, Ferric chelate reductase 1; FOXM1, Forkhead Box M1; FZD4, frizzled family receptor 4; H3K4me3, histone H3 lysine 4 tri; PIK3R3, Phosphoinositide-3-Kinase Regulatory Subunit 3; WDR5, WD Repeat Domain 5).

Prostate cancer is the cancer with the highest incidence among men globally. Distant metastasis and castration-resistant prostate cancer (CRPC) are the main causes of death for prostate cancer patients (Schaeffer et al., 2024). Some studies have found by detecting HOXD-AS1 in the serum exosomes of patients with PC that HOXD-AS1 can promote the metastasis of PC by regulating the miR-361-5p/Forkhead Box M1 (FOXM1) axis through the ceRNA mode. Moreover, the upregulation of HOXD-AS1 is positively correlated with lymph node metastasis in PC patients. ROC curve analysis evaluates the diagnostic potential of serum exosomal HOXD-AS1 for distant metastasis (AUC = 0.797). Also, the creation of a mouse bone metastasis model further proved that exosomal HOXD-AS1 from CRPC cells can promote the distant metastasis of PC cells. The detection of exosomal lncRNA is a non-invasive biopsy with high compliance and can be sampled and detected repeatedly. It has high research value and feasibility. This study shows that serum exosomes can be used as a biomarker for diagnosing metastatic PC and is a promising liquid biopsy biomarker (Jiang et al., 2021). Moreover, *in vitro* studies showed that knocking down HOXD-AS1 stops CRPC cell growth and division. By mediating H3 lysine four trimethylation (H3K4me3) to regulate WD Repeat Domain 5 (WDR5) expression, it inhibits CRPC cell proliferation and migration. Also, the nude mouse subcutaneous tumor model proved that HOXD-AS1 knockdown can shrink CRPC tumor size and lower their chemo-resistance, especially with paclitaxel treatment. This not only provides a new perspective for the study of CRPC regulation by lncRNA HOXD-AS1 but also offers a potential approach for the treatment of CRPC (Gu et al., 2017). Table 2 and Figure 2 summarizes the biological functions and regulatory mechanisms of HOXD-AS1 in genitourinary system tumors.

**TABLE 2:** The biological function and regulatory mechanism of HOXD-AS1 in urogenital system tumors.

Cancer types	Expression	Sample type	Clinical features	Biological functions	Related genes and pathways	References
Ovarian Cancer	Upregulated	Tissues and cell lines	Overall survival and disease-free survival, metastasis, FIGO stage Pathological grades Lymph node metastasis	Proliferation (+), Migration (+), Invasion (+)	miR- 608/FZD4, miR-186-5p/PIK3R3	Dong et al. (2019), Wang et al. (2018)
Cervical Cancer	Upregulated	Cell lines	Overall survival and disease-free survival. Metastasis, Lymphovascular invasion, tumor volume, Lymph node metastasis, TNM stage, Recurrence	Proliferation (+),Migration (+), Invasion (+)	ELF1/FRRS1 miR-877-3p/FGF2,	Chen and Li (2020), Liu et al. (2022)
Prostate Cancer	Upregulated	serum	Overall survival, disease-free survival, Lymph node Metastasis, Distant Metastasis, Tumor stage, Exosomal HOXD-AS1, tumor volume	Proliferation (+), Migration (+), Invasion (+), Drug resistance (+), Castration resistance (+)	miR-361-5p/FOXM1, H3K4me3/WDR5	Jiang et al. (2021), Gu et al. (2017)

## 5 Other cancers

### 5.1 Thyroid cancer

Thyroid cancer is the most common malignant tumor among adolescents and adults aged 16 to 33 (Chen et al., 2023). Some studies have conducted differential analysis of the whole-genome expression profiles of thyroid tissues from 18 TC patients and four healthy participants. Among the five risk genes, lncRNA HOXD-AS1 was found to be upregulated in thyroid cancer tissues, and the expression of HOXD-AS1 was significantly correlated with the clinical stage of TC. The high expression of HOXD-AS1 was also associated with age (60 vs. ≥ 60, *P* = 0.012), tumor size (≤4 cm vs. > 4 cm, *P* = 0.014), and lymph node metastasis (NO vs. YES, *P* < 0.001) (Xu and Feng, 2019; Du et al., 2017). However, in the TCGA database, the expression of HOXD-AS1 has no significant correlation with the disease-free survival and overall survival of patients therefore, whether HOXD-AS1 can become a biomarker for predicting the clinical progression of thyroid cancer warrants further research and confirmation.

### 5.2 Breast cancer

According to the World Health Organization, the incidence of breast cancer (BC) has surpassed that of lung cancer to become the cancer with the highest incidence (Sung et al., 2021). HOXD-AS1 is upregulated in BC tissues and promotes tumor progression. In various BC cell lines such as MDA-MB-435 and MCF-7, there is a causal relationship between the high expression of HOXD-AS1 and cell biological behaviors such as cell cycle regulation, proliferation, epithelial-mesenchymal transition, migration, and invasion. Specifically, HOXD-AS1 acts as a competitive binding to miR-421 to regulate the Sox4-related signaling pathway of the target gene and plays a regulatory role in the occurrence of the above cell biological behaviors (Li et al., 2019). Therefore, the HOXD-AS1/miR-421/SOX4 axis may be a new target for the treatment of breast cancer patients.

### 5.3 Lung cancer

Lung cancer (LC) is the leading cause of cancer-related death worldwide, and Non-small cell lung cancer (NSCLC) is the dominant tissue subtype of LC (Leiter et al., 2023). Studies have shown that HOXD-AS1 is specifically upregulated in NSCLC tissues (*P* < 0.001) and promotes the growth of cancer cells by targeting miR-147a. The expression of HOXD-AS1 was correlated with the clinicopathological features of NSCLS (tumor size, *P* = 0.006; tumor staging, *P* = 0.044; recurrence (*P* = 0.031) and survival rate (*P* = 0.003) were positively correlated (Wang Q. et al., 2017). Additionally, the expression of HOXD-AS1 in the nucleus is downregulated in lung adenocarcinoma tissues, and its high expression has a tumor-inhibitory effect and is related to a better prognosis (Guo et al., 2019). Studies have shown that HOXD-AS1 may play different roles in various human cancers.

### 5.4 Glioma

Gliomas are heterogeneous tumors originating from glial cells and represent the most common type of brain tumor (Laug et al., 2018). Kaplan-Meier survival analysis indicated that glioma patients with high HOXD-AS1 expression had a poor prognosis (*P* = 0.0036) (Chen et al., 2018; Zhou et al., 2019). Regarding the mechanism, HOXD-AS1 binds to miR-130 a and miR-204 to regulate the transcription factor E2F Transcription Factor 8 (E2F8) related signaling pathway through ceRNA mode and plays a regulatory role in the occurrence of the above cell biological behaviors. At the same time, HOXD-AS1 can also participate in RA signal transduction through PI3K/AKT and MAPK/Erk pathways, promoting tumor angiogenesis and inflammatory response (Chen et al., 2018; Zhou et al., 2019). In conclusion, these studies suggest that HOXD-AS1 provides a promising therapeutic target for glioma patients.

### 5.5 Neuroblastoma

Neuroblastoma is an aggressive tumor that occurs more frequently in children and has a poor prognosis. In high-risk cases, the 5-year overall survival probability is approximately 50%. The high expression of HOXD-AS1 is associated with the poor prognosis of patients with neuroblastoma, suggesting that HOXD-AS1 may be a potential diagnostic and prognostic marker for neuroblastoma (Yarmishyn et al., 2014).

### 5.6 Melanoma

**FIGURE 3:**
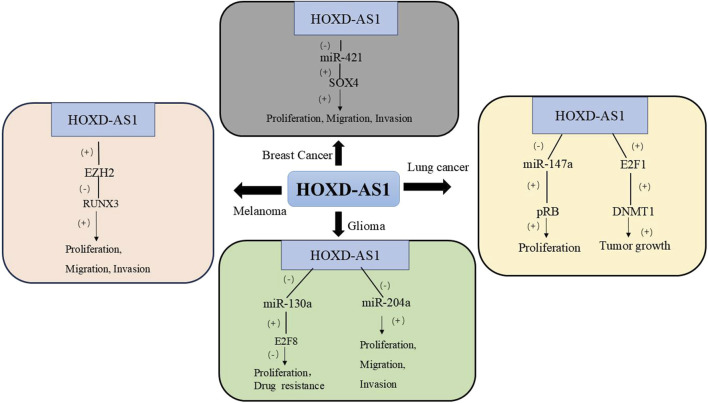
The regulatory network of HOXD-AS1 on other tumors. (DNMT1, DNA Methyltransferase 1; EZH2, Enhancer of Zeste Homolog 2; E2F1, E2F Transcription Factor 1; pRB, Retinoblastoma protein; RUNX3, Runt-related Transcription Factor 3; SOX4, SRY-related HMG-box 4; E2F8, E2F Transcription Factor 8).

Melanoma is the most aggressive form of skin cancer and its incidence has been increasing globally in recent years (Siegel et al., 2023a). According to clinical data, the expression of HOXD-AS1 in melanoma tissues is upregulated, which is related to the lower survival time of melanoma patients. The expression of HOXD-AS1 in melanoma tissues is upregulated, which is associated with lower survival in melanoma patients. In various melanocyte lines such as A375, A2598, and B16, high expression of HOXD-AS1 can promote the proliferation and invasion of melanoma cells. Mechanistically, HOXD-AS1 acts by epigenetically inhibiting the expression of dwarf-associated Runt-related Transcription Factor 3 (RUNX3) by binding to Enhancer of Zeste Homolog 2 (EZH2) (Zhang et al., 2017). Table 3 and Figure 3 summarizes the biological functions and regulatory mechanisms of HOXD-AS1 in other tumors.

**TABLE 3:** The biological function and regulatory mechanism of HOXD-AS1 in other tumors.

Cancer types	Expression	Sample type	Clinical features	Biological functions	Related genes and pathways	References
Thyroid Cancer	Upregulated	Tissues	Age, Tumor size, lymph node metastasis, Distant metastasis	Proliferation	—	Xu and Feng (2019), Du et al. (2017)
Breast Cancer	Upregulated	Tissues and cell lines	TNM stage, Distant metastasis, Overall survival, tumor volume	Proliferation (+), Migration (+), Invasion (+), Apoptosis (−)	miR-421/SOX4	Li et al. (2019)
Lung cancer	Upregulated	Tissues	Overall survival TNM stage, tumor volume, Recurrence status, Lymph node metastasis	Proliferation (+)	miR-147a/pRB	Wang et al. (2017b)
Lung cancer	Down	Tissues		Tumor growth (+),	E2F1/DNMT1	Guo et al. (2019)
Glioma	Upregulated	Tissues and cell lines	Overall survival, tumor volume	Proliferation (+), Drug resistance (+) Migration (+), Invasion (+)	miR-130a/E2F8, miR-204a	Chen et al. (2018), Zhou et al. (2019)
Melanoma	Upregulated	Tissues and cell lines	Overall survival, tumor volume	Proliferation (+), Migration (+), Invasion (+)	EZH2/RUNX3	Zhang et al. (2017)

## 6 Conclusion

As the research on lncRNAs in tumors deepens, the role of lncRNAs in tumor occurrence and development is worthy of further exploration and summary. Current studies have indicated that HOXD-AS1 is a carcinogenic lncRNA. It is upregulated in multiple cancer tissues and cell lines and is associated with poor prognosis of patients, being regarded as a potential biomarker and therapeutic target for the prognosis of multiple tumors.

In terms of expression and mechanism, HOXD-AS1 is often highly expressed in cancers like hepatocellular, gastric, and ovarian cancers. It promotes tumor cell growth, blocks apoptosis, and aids migration and invasion. Many studies show HOXD-AS1 acts as a ceRNA, regulating target genes by adsorbing miRNA and fueling tumor development. In HCC, knocking down HOXD-AS1 cuts B-cell lymphoma-2 (Bcl-2) (anti-apoptotic) and boosts Bcl-2 Associated X protein (Bax) (pro-apoptotic), triggering apoptosis (Sun et al., 2020). In colorectal cancer, it targets miR-217 to upregulate Bcl-2 and stop apoptosis (Li X. et al., 2018). In ovarian cancer, it binds miR-186-5p to regulate the PIK3R3 pathway and EMT proteins, promoting EMT (Wang et al., 2018). In cholangiocarcinoma, it binds miR-520c-3p to regulate MYCN, aiding cell migration, invasion, and EMT (Li et al., 2020). Overall, HOXD-AS1 regulates cell growth, migration, invasion, and apoptosis in various cancers, sometimes *via* protein expression or as a ceRNA. But in different cancers, the molecules, pathways, miRNAs, and target genes it involves vary, affecting how it controls tumor cell behavior.

In cell biology, HOXD-AS1 regulates by affecting the interaction between microRNA and target gene’s 3′-UTR. This causes messenger RNA demethylation and instability, impacting tumor initiation and progression. The subcellular location of HOXD-AS1 leads to different signaling pathways for tumor development, and its downstream tumor-influencing factors vary. In CRC, low nuclear HOXD-AS1 expression means poor prognosis. HOXD-AS1 recruits PRC2, causing H3K27 methylation on HOXD3 promoter to inhibit HOXD3 transcription. Then, HOXD3 activates integrin β3 subunit transcription, triggering the MAPK/AKT pathway (Yang et al., 2019). In HCC, HOXD-AS1 is mainly in the cytoplasm. It binds miR-130a-3p to stop SOX4 degradation, activating EZNH2 and MMP2 expression and aiding HCC metastasis (Wang H. et al., 2017). In cancers like cholangiocarcinoma, cervical and ovarian cancer, HOXD-AS1 dysregulation promotes tumor growth. But its subcellular location’s role is unclear, and research on its relation to cell death like autophagy and ferroptosis is scarce. Deeper study is needed.

According to clinical research, lncRNAs can be easily identified in various biological specimens including tissue samples, saliva, or plasma, and exhibit certain cell specificity or stage specificity, making it possible for HOXD-AS1 to serve as a biomarker in the future. Currently, RT-QPCR technology is mostly used for serum exosome detection. We can also achieve higher sensitivity and reliability by combining RT-QPCR with next-generation sequencing technology (NGS). Thirdly, drug use for tumor patients is also a key focus of clinical research. There are an increasing number of related treatments such as chemotherapy, immunotherapy, and targeted drug use. However, there are still relatively few studies on the mechanism of HOXD-AS1 participating in tumor drug resistance. It is only simply proposed that it can regulate the related signaling pathways of target genes through the ceRNA mode. Further exploration of upstream and downstream signals and the specific molecular regulatory network downstream is needed. In conclusion, HOXD-AS1 is a crucial tumor-influencing factor and a therapeutic target and biomarker with promising clinical application prospects for early diagnosis and prognosis.

## Glossary

AIG-1: Apoptosis-Inducing Gene 1
ARHGAP11A: Rho GTPase activating protein 11A
BC: breast cancer
Bax: Bcl-2 Associated X protein
Bcl-2: B-cell lymphoma-2
CC: cervical cancer
CCA: cholangiocarcinoma
CCND1: Cyclin D1
CRC: colorectal cancer
CRPC: Castration-resistant prostate cancer
c-Myc: MYC proto-oncogene
DNMT1: DNA Methyltransferase 1
EZH2: Enhancer of Zeste Homolog 2
EZNH2: Enhancer of Zeste Homolog 2
E2F1: E2 Factor 1
E2F8: E2F Transcription Factor 8
HBx: HBV protein X
FGF2: fibroblast growth factor 2
FOXM1: Forkhead Box M1
FRRS1: Ferric chelate reductase 1
FZD4: frizzled family receptor 4
GC: gastric cancer
GCO: global cancer observatory
HCC: hepatocellular carcinoma
HBV: Hepatitis B Virus
HCV: Hepatitis C Virus
H3K4me3: histone H3 lysine four tri
JAK2: Janus kinase 2
LC: lung cancer
LDHA: lactate dehydrogenase A
lncRNAs: Long non-coding RNAs
MYCN: V-Myc Avian Myelocytomatosis Viral Oncogene Neuroblastoma Derived Homolog
NSCLC: Non-small cell lung cancer
OC: ovarian cancer
OS: overall survival
OSCC: oral squamous cell carcinoma
PC: pancreatic cancer
PFS: progression-free survival
PIK3R3: Phosphoinositide-3-Kinase Regulatory Subunit 3
PLAC8: Placenta Specific Protein 8
pRB: retinoblastoma protein
ROC: receiver operating characteristic
RT-qPCR: reverse transcription-polymerase chain reaction;
RUNX3: Runt-related Transcription Factor 3
SLC27A4: Solute Carrier Family 27 Member 4
SOX4: SRY-related HMG-box 4
STAT3: Signal Transducer and Activator of Transcription 3
TC: thyroid cancer
TNM: tumor node metastasis
WDR5: WD Repeat Domain 5.
